# A smoothing and bootstrap-based framework for early outbreak detection

**DOI:** 10.1371/journal.pone.0345088

**Published:** 2026-03-23

**Authors:** Lengyang Wang, Yingcun Xia, Ee Hui Goh, Mark Chen

**Affiliations:** 1 Advanced Methods and Analytics, Communicable Diseases Agency, Singapore, Singapore; 2 Department of Statistics and Data Science, National University of Singapore, Singapore, Singapore; 3 Department of Epidemiology and Preventive Medicine, Tan Tock Seng Hospital, Singapore, Singapore; The University of New Mexico, UNITED STATES OF AMERICA

## Abstract

Timely detection of infectious disease outbreaks is critical for effective public health response. The effective reproduction number (*R*_*t*_) is a key metric that captures transmission dynamics and signals the potential onset of outbreaks when it rises above 1. However, day-of-the-week and public holiday effects, along with random fluctuations in reported cases, can distort *R*_*t*_ estimates and reduce their usefulness for real-time surveillance. In this study, we present an *R*_*t*_-based outbreak detection framework that integrates calendar-aware smoothing with bootstrap inference to quantify the uncertainty of smoothed *R*_*t*_ estimates. Using daily COVID-19 case data from Singapore, we evaluate several smoothing approaches—including a working-day moving average (MAH) that adjusts for public holidays—and compare the performance of the proposed method with established outbreak detection algorithms such as Early Aberration Reporting System (EARS), Bayesian-based detection methods (EpiEstim) and logistic regression–based approaches. In our framework, calendar-aware smoothing is not a generic pre-processing choice but a necessary, model-agnostic step that produces *R*_*t*_ inputs with reduced calendar artefacts. This makes subsequent inference and testing on *R*_*t*_ both more stable and more interpretable. Our results show that smoothing, particularly with MAH, improves the stability of *R*_*t*_ estimates and enables more reliable outbreak detection. The proposed method consistently demonstrates superior timeliness across observed and simulated outbreaks, while maintaining desired false positive rates. Simulation studies further confirm its robustness under varying sample sizes and case volumes, highlighting advantages over other methods. In conclusion, the proposed method offers a simple, interpretable, and theoretically grounded framework for early outbreak detection. Its consistent performance across real and simulated data suggests it may be broadly applicable to other infectious diseases with similar transmission dynamics.

## 1 Introduction

Surveillance of infectious diseases intends to provide insights into the progression and impact of epidemics. This is essential for effective planning and mitigation strategies for the population [1]. An early warning model for infectious diseases is a crucial tool for timely monitoring, prevention, and control of disease outbreaks [2] and hence enable rapid implementation of control measures to minimize their impact on public health [3]. In the context of an evolving epidemic with a significant number of infected individuals, the effective reproduction number (*R*_*t*_) is a crucial metric based on the underlying principles of transmission dynamics. It considers the size of the remaining susceptible population and encapsulates various factors affecting the epidemic’s trajectory [4]. For the purpose of monitoring COVID-19 outbreaks, daily reported cases can be utilized to estimate *R*_*t*_. An outbreak would continue and case numbers should increase when *R*_*t*_ > 1, but it would subside when *R*_*t*_ < 1 [5,6]. Theoretically, when there is switch from *R*_*t*_ < 1 to *R*_*t*_ > 1, either due to changes in the virus, population level immunity, or the interactions within an at-risk population, we should anticipate that an outbreak is imminent. However, there are several challenges in reliably estimating *R*_*t*_ from available data sources [7].

One key artefact in healthcare data used for surveillance are day-of-the-week and public holiday effects [8,9]. For instance, many healthcare facilities, particularly outpatient and primary care clinics, are typically closed on Sundays and public holidays. This closure results in a sharp decline in reported cases on those days, followed by a surge on the first working day after, leading to misleading troughs and spikes that do not accurately reflect the trajectory of an epidemic. If these systematic day-of-the-week and public holidays effects are inadequately accounted for, they can obscure genuine increases as well as cause artefactual post-weekend and post-holiday increases in case notifications, both of which can adversely affect the estimation of *R*_*t*_, and render *R*_*t*_ less useful as a tool to support timely decision-making. Methods to adjust for day-of-the-week and public holidays effects have been evaluated [8], but not specifically for adjusting and monitoring estimates of *R*_*t*_.

However, even after accounting for day-of-the-week and public holiday effects, we would still face the challenge of determining when *R*_*t*_ has truly switched to a value above 1. Counts of cases exhibit stochastic fluctuations around a mean that reflects some baseline incidence. Just on the basis of sampling variation, we could observe a relative increase in cases and hence *R*_*t*_ > 1 because illness episodes presenting at sentinel clinics or those selected for testing by chance included more infections than in a preceding period. We must thus determine when *R*_*t*_ is significantly above 1 before *R*_*t*_ can be used for predicting when an outbreak is imminent.

In this work, we first focused on evaluating calendar-aware smoothing techniques [10] that account for day-of-the-week and public holiday effects, to assess how well these methods work with case notification data for COVID-19 from Singapore, and which are most appropriate when estimating of *R*_*t*_. Building on these results, we integrated these smoothing approaches into an *R*_*t*_-based outbreak detection framework designed to provide robust and timely signals despite random fluctuations in reported cases. The framework incorporates calendar-aware smoothing as a core component, ensuring that *R*_*t*_ estimation and bootstrap inference are based on data adjusted for calendar-related artefacts. This integration strikes a balance between interpretability and simplicity, yielding stable outbreak signals with low false-positive rates (fpr) – features that support more responsive and efficient public health action. We then demonstrate the validity of these methods for detecting a series of COVID-19 outbreaks from case notification data in Singapore.

## 2 Materials and methods

### 2.1 Data

In this study, we analysed daily reported COVID-19 cases in Singapore from 2021/04/01–2023/2/13, when COVID-19 diagnoses were routinely notified to the Ministry of Health. This period encompassed significant outbreaks driven by variants such as Delta, BA.1/2, BA.4/5, and XBB, which were used to assess the effectiveness of various data smoothing and outbreak detection techniques [11]. By integrating smoothing methods with the estimation of *R*_*t*_ and bootstrap techniques, we compared our proposed outbreak detection method against existing approaches during key outbreak periods. Furthermore, we conducted a simulation study focusing on the BA.4/5 and XBB subvariants to explore how the total number of daily observed cases, and the sample size of individuals tested to ascertain the variant could affect outbreak detection.

The replacement of previously dominant variants by emerging ones was monitored using whole genome sequencing (WGS), the gold standard for identifying and classifying SARS-CoV-2 variants. WGS enables both the detection of known lineages and the surveillance of new ones, making it essential for tracking viral evolution and informing public health strategies [12].

### 2.2 Methodological framework

#### 2.2.1 Calendar-aware smoothing method.

Calendar-related anomalies introduce systematic trough–spike patterns in reported case counts. Because *R*_*t*_ can be expressed as a nonlinear function (e.g., a ratio) of short-term weighted sums of past incidence [13], these irregularities can be amplified by *R*_*t*_ estimators and obscure early outbreak signals. To address this, our framework includes calendar-aware smoothing as a required Step 1, producing incidence series whose short-horizon aggregates remain comparable across weeks regardless of weekend/holiday placement. Subsequent *R*_*t*_ estimation and bootstrap testing are then applied to these calendar-stabilized series.

A 7-day moving average (MA) is the simplest calendar-aware smoothing approach to remove the day-of-the-week effect. For example, the daily cases after MA smoothing are calculated as:


$$Y_{t}^{\prime }=\frac{Y_{t}+Y_{t-1}+\cdots +Y_{t-6}}{7}.$$

However, a drawback of this method is its inability to account for public holiday effects. The working-day moving average method, which treats both public holidays and weekends as non-working days and the remaining days as working days (denoted as MAH), as described in [8], addresses this limitation. Let *T* represent the number of working days within a given 7-day block. In this method, the calculation involves:

1. Multiplying the sum of cases on working days by $\frac{5}{T}$.
2. Multiplying the sum of cases on non-working days by $\frac{2}{7-T}$.

This adjustment mirrors the typical operations of most primary care clinics, which operate five days a week during non-public holiday periods. The overall smoothed cases are calculated by dividing the sum of these adjusted totals by 7. For consecutive 7-day blocks without public holidays, this approach yields results identical to the MA method. However, when public holidays are included, the MAH method marginally increases the weight of working days while reducing that of non-working days, to make the data for that period more comparable to a typical 7-day block without public holidays.

[8] also described an extended working day moving average method that utilizes historical data to calculate specific scaling factors for each day of the week. While this approach can provide more accurate estimates, its reliance on historical data limits its applicability to short data periods. As a result, we have opted not to include this method in our analysis.

#### 2.2.2 Estimation of *R*_*t*_ based on observed data.

Prior to presenting our proposed method for outbreak detection, we outline a technique based on observed data with a fixed generation time (time between the infection of a primary case and one of its secondary cases) of H days for estimating *R*_*t*_ [14]. Assuming a fixed generation time of H = 3 [15], the *R*_*t*_ is determined by taking the sum of new reported cases over a span of 3 consecutive days and comparing it to the sum of new reported cases from the 3 days preceding that period. For instance, let’s illustrate this with some hypothetical data in Table 1:

**Table 1:** New Reported Cases Over Days.

Day 1	Day 2	Day 3	Day 4	Day 5	Day 6
10	20	30	40	50	60
		**60**			**150**

Now, for Day 6, the estimated $R_{t}=\frac{\text{Sum of new reported cases from days 4-6}}{\text{Sum of new reported cases from days 1-3}}=2.5.$

For instance, let’s illustrate this with some hypothetical data:

#### 2.2.3 Bootstrap method for drawing statistical inference.

We use a bootstrap method [16–18] to draw statistical inference for *R*_*t*_, since accurate estimation of the asymptotic distribution of *R*_*t*_ is challenging. In this analysis, we considered two scenarios:

**Scenario (a)** involved examining the total observed infections notified to health authorities. This included both cases diagnosed by PCR-based assays and Rapid Antigen Tests, which do not distinguish between variants.
**Scenario (b)** involved a stratified analysis for a new variant versus the incumbent variant, including only a subset of total cases tested by PCR-based assays with differentiation between variants using SGTF (and potentially WGS, although this introduces a lag due to turnaround time).

Let *Y*_*t*_ represent the daily COVID-19 cases and assume $Y_{t}∼\text{Poisson}(\lambda_{t})$. We assume the data-generating process (DGP) belongs to a parametric class ${ℳ}_{n}(Y_{t-1},Y_{t-2},\ldots ,Y_{t-p})$, such that:


$$𝔼(Y_{t}|Y_{t-1},Y_{t-2},\ldots ,Y_{t-p})={ℳ}_{n}(Y_{t-1},Y_{t-2},\ldots ,Y_{t-p}).$$

Denote $\hat{{ℳ}_{n}(\cdot )}$ as the estimator of ${ℳ}_{n}(\cdot )$, which we specify as an ARIMA model to estimate the conditional mean $\lambda_{t}$. Let $Y_{t}^{\left(d\right)}=(1-B{)}^{d}Y_{t}$ denote the *d*-th order differenced series. Then we have:


$$𝔼(Y_{t}^{\left(d\right)}|Y_{t-1}^{\left(d\right)},Y_{t-2}^{\left(d\right)},\ldots ,Y_{t-p}^{\left(d\right)})=c+\sum_{i=1}^{p}\phi_{i}Y_{t-i}^{\left(d\right)}+\sum_{j=1}^{q}\theta_{j}\varepsilon_{t-j},$$

in which *B* is lag operator, $\varepsilon_{t}$ is the error term and *p*,*d*,*q* are selected by the model with the smallest AIC. The final inference and forecasts are mapped back to the original scale by applying the inverse differencing transformation.

For scenario (a), with observed COVID-19 cases $\{Y_{t}{\}}_{t=1}^{n}$, define bootstrap samples $\{Y_{t}^{*}{\}}_{t=1}^{n}$ by:


$$Y_{t}^{*}∼\text{Poisson}\left(\hat{{ℳ}_{n}}(Y_{t-1},Y_{t-2},\ldots ,Y_{t-p})\right).$$ (1)

The advantage of this design is that it preserves and mimics the dependence structure of the underlying observations, making it well-suited for analysing time series data [16].

For **scenario (b)**, let *V*_*t*_ denote the number of infections due to a variant of concern (VOC) among *S*_*t*_ people tested, with the corresponding proportion $P_{t}=V_{t}/S_{t}$. The estimated total daily cases of the VOC are $I_{t}=Y_{t}\times P_{t}$. Similarly, we assume:


$$V_{t}∼\text{Poisson}(\hat{\Gamma_{n}}(V_{t-1},V_{t-2},\ldots ,V_{t-p})),$$

where $\Gamma_{n}(\cdot )$ serves a role analogous to ${ℳ}_{n}(\cdot )$, representing a time series model (e.g., ARIMA) that estimates the conditional mean of *V*_*t*_ based on its historical values.

Then, with observed $\{Y_{t}{\}}_{t=1}^{n}$, $\{S_{t}{\}}_{t=1}^{n}$, and $\{V_{t}{\}}_{t=1}^{n}$, define bootstrap samples $\{I_{t}^{*}{\}}_{t=1}^{n}$ by:


$$I_{t}^{*}=\frac{\text{Poisson}\left(\hat{{ℳ}_{n}}(Y_{t-1},Y_{t-2},\ldots ,Y_{t-p})\right)\times \text{Poisson}\left(\hat{\Gamma_{n}}(V_{t-1},V_{t-2},\ldots ,V_{t-p})\right)}{S_{t}}.$$ (2)

Note that $\{Y_{t}{\}}_{t=1}^{n}$ can be either the original observed cases or the adjusted values after accounting for day-of-week and public holiday effects, as described in Section 2.2.1. However, based on our results, we strongly recommend applying calendar-aware smoothing when calendar anomalies are present.

Next, at time *t*, we compute the effective reproduction number *R*_*t*_ as described in Section 2.2.2. To draw statistical inference for *R*_*t*_, we perform bootstrapping on the time series from t−2H+1 to t−H, using either (1) or (2), and repeat this *B* times.

For scenario (a), denote the bootstrap effective reproduction number at time *t* by:


$$R_{t}^{\left(b\right)}=\frac{\sum_{s=t-2H+1}^{t-H}Y_{s}^{*}}{\sum_{s=t-2H+1}^{t-H}Y_{s}},\;b=1,2,\ldots ,B,$$

and for scenario (b), denote:


$$R_{t}^{\left(b\right)}=\frac{\sum_{s=t-2H+1}^{t-H}I_{s}^{*}}{\sum_{s=t-2H+1}^{t-H}I_{s}},\;b=1,2,\ldots ,B.$$

#### 2.2.4 Outbreak detection algorithm summary.

Combining the previous methods, our proposed approach for outbreak detection can be described in the following steps.

We illustrate the procedure using **Scenario (a)**. Given observed cases $\{Y_{t}{\}}_{t=1}^{n}$, we aim to test the hypothesis:


$$H_{0}:\text{There is no outbreak signal at time }t,\text{ i.e., }R_{t}\leq 1.$$

**Step 1: Calendar-aware smoothing.** Mitigate the day-of-the-week and public holiday effects in the observed cases using the methods described in Section 2.2.1. Denote the smoothed case series as $\{Y_{t}^{\prime }{\}}_{t=1}^{n}$. This step is essential to the framework, as all subsequent *R*_*t*_ estimation and inference are performed on the calendar-adjusted series *Y*_*t*_’ to ensure that short-term fluctuations are not confounded by calendar effects.

**Step 2: Compute *R***_***t***_**.** At time *t*, compute the effective reproduction number *R*_*t*_ from the smoothed case series, assuming a constant generation interval *H*, as described in Section 2.2.2.

**Step 3: Bootstrap under *H***_**0**_**.** Generate bootstrap samples $\{Y_{t}^{\prime *}{\}}_{t=1}^{n}$ under the null hypothesis *H*_0_ using the approach in Section 2.2.3. From time t−2H+1 to t−H, compute the bootstrap effective reproduction number at time *t* as:


$$R_{t}^{\left(b\right)}=\frac{\sum_{s=t-2H+1}^{t-H}Y_{s}^{\prime *}}{\sum_{s=t-2H+1}^{t-H}Y_{s}^{\prime }},\;b=1,2,\ldots ,B.$$

**Step 4: Hypothesis testing.** Calculate the p-value at time *t* as:


$$p_{t}=\frac{B-\sum_{b=1}^{B}𝕀(R_{t}>R_{t}^{\left(b\right)})}{B},$$

where 𝕀(·) is the indicator function. We reject the null hypothesis *H*_0_ if p<α for all p∈𝒫, where:


$$𝒫=\{p_{t-K+1},\ldots ,p_{t}\}.$$

The parameters *K*, *H*, and *α* are selected to ensure that the fpr over the entire monitoring period remains below a prespecified level. Further details on parameter calibration are provided in the following section.

This four-step procedure combines calendar-aware smoothing and bootstrap inference within an *R*_*t*_-based structure, producing epidemiologically interpretable and statistically supported outbreak signals.

### 2.3 Statistical comparison of different detection methods

Daily reported COVID-19 cases in Singapore from 2021/04/01 to 2023/2/13, were used to compare smoothing and outbreak detection methods. The smoothing approaches used in the proposed outbreak detection framework were:

- **No smoothing (NS)**
- **7-day moving average (MA)**
- **Working-day moving average (MAH)**

We also compared our proposed outbreak detection method, referred to as **PM**, with the following:

- **Early Aberration Reporting System (EARS)** [19]: We implemented all three EARS methods (C1–C3). In EARS C1, an outbreak signal is detected if the current observed value exceeds the mean of the previous *w* reporting periods plus the product of a critical value (corresponding to significance level *α*) and the standard deviation. C2 differs by shifting the *w*-day baseline window to the left with a gap of *g* = 3 days. While C1 and C2 are analytically equivalent in the absence of outbreaks, C2 is more sensitive to continued outbreaks. C3 modifies C2 by using a partial sum of positive daily deviations over the current and previous two days [20]. For analysis, we use the abbreviation **EC**.
- **Bayesian-based detection [13]**: The EpiEstim method, introduced by [13], provides a framework for estimating the time-varying effective reproduction number (*R*_*t*_) during an epidemic using daily incidence data and the serial interval distribution. It relies on a renewal equation approach, assuming that new cases arise from past infections weighted by the serial interval. The method adopts a Bayesian formulation, allowing for real-time estimation of *R*_*t*_ with uncertainty quantified via posterior distributions. We define an outbreak signal at time *t* if the lower bound of the estimated *R*_*t*_ exceeds 1. For analysis, this method is referred to using the abbreviation **EPI**.
- **Logistic regression-based detection** [21,22]: These methods estimate *R*_*t*_ of a new variant of concern (VOC) relative to the incumbent variants using logistic regression. Suppose the VOC variant *v* is a specific value of the sequence *S*_*t*_, and *T*_*t*_ is the sampling time. Then, the logistic regression model is:

$$\mathbb{P}(S_{t}=v)=\frac{\exp(\beta_{0,v,t}+\beta_{1,v,t}T_{t})}{1+\exp(\beta_{0,v,t}+\beta_{1,v,t}T_{t})},$$

where $\beta_{1,v,t}$ represents the growth rate difference between the VOC and the incumbent variants at time *t*. While *R*_*t*_ can be derived using generation time dis*t*ributions, these can vary across regions and time periods. Thus, we treat $\beta_{1,v,t}$ as the outbreak signal and define an outbreak as occurring at time *T*_*i*_ if $\beta_{1,v,t}>0$ with statistical significance at level *α*. This method is abbreviated as **Logi** in our analysis.

Other commonly used methods for outbreak detection are the Farrington algorithm [23] and CUSUM [24]. However, both may be suboptimal for COVID-19 surveillance. The Farrington algorithm is designed primarily for diseases with clear seasonal and periodic patterns and typically relies on over a year of historical baseline data. In contrast, CUSUM requires careful parameter tuning for each outbreak, as different outbreak magnitudes necessitate different threshold settings. A single set of parameters cannot accommodate all scenarios, and multiple parameter combinations may yield similar false positive rates. This complicates fair comparisons of detection timeliness, as performance may be heavily influenced by the chosen parameter combination.

Suppose the detection period of an epidemic starts at *t*_1_ and ends at *t*_2_. We computed the following performance metrics for comparison:

- **Timeliness** = $\frac{t_{2}-(\text{time of first outbreak signal})}{t_{2}-t_{1}}$,
- **False positive rate (fpr)** = $\frac{\text{Total number of detected signals}}{t_{2}-t_{1}}$.

**Fig 1:**
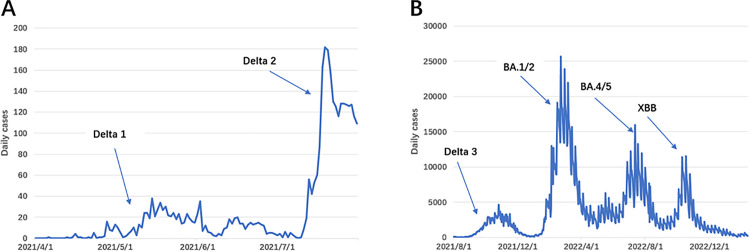
Fig A and B indicate outbreak caused by different varian*ts.*

For timeliness, the testing period extended from the onset of a new epidemic until its peak. As shown in Fig 1, for the Delta variant, we identified three major outbreaks:

- An outbreak arising at Changi Airport [25],
- An outbreak arising at Jurong Fishery Port [26],
- A widespread outbreak following the relaxation of COVID-19 restrictions [27].

For other variants, major outbreaks were identified due to several omicron subvariants, with specific dates detailed in the S1 Supplementary Materials. For the false positive rate, the testing periods were defined as the intervals between the peak of a previous epidemic and the onset of a new one. During these non-epidemic periods, any detected outbreak signal is considered a false positive.

#### 2.3.1 Simulation analysis 1.

Determining an appropriate sample size for testing is critical to the effectiveness of surveillance systems, as it directly influences data analysis and the system’s ability to achieve public health objectives [28], while being financially and logistically feasible. To address this, we evaluated how varying the total daily observations and sample sizes of tested individuals affects the timeliness of different methods. Our simulation study focused on the BA.4/5 and XBB variant waves, using available data for analysis [11,29]. It is important to note that this analysis was designed to assess the sensitivity of each method to changes in observation scale and testing sample size, rather than to directly compare timeliness across different methods.

For the BA.4/5 variant wave, the logistic regression method treated both BA.2 and BA.2.10 as the incumbent strains. In this case, binary logistic regression was applied, with BA.2 and BA.2.10 combined as the reference group. In contrast, during the XBB wave, the incumbent strains included the BA.2.75, BA.4/5, and BA.2. Given the presence of four variants, multinomial logistic regression was employed. Both BA.2.75 and BA.4/5 were considered as potential reference groups, and the results from the logistic regression method were averaged to account for both scenarios.

Owing to methodological and data structure limitations, we were unable to access the false positive rate for the logistic regression method. We hence simply fixed its significance level at 5%. For the other methods, we used the same parameter settings as in the previous analysis, which compared the performance of each method using observed COVID-19 data from Singapore.

In the simulation:

- Let $\{Y_{t}{\}}_{t=1}^{n}$ represent the simulated daily COVID-19 cases, modeled by $\hat{{ℳ}_{n}}(Y_{t-1},Y_{t-2},\ldots ,Y_{t-p})$, where $\hat{{ℳ}_{n}}(\cdot )$ is an ARIMA model fitted to the observed cases during the BA.4/5 or XBB outbreaks.
- Let $Y_{t}\times a$ represent the adjusted daily COVID-19 observations, where a∈{0.25,0.5,1,2,3}. This adjustment examines the impact of total daily observations.
- Denote the daily sample size of tested individuals as $Y_{t}\times a\times b$, where b∈{0.05,0.1,0.25,0.5}. This adjustment examines the impact of the sample size of tested individuals.

**Fig 2:**
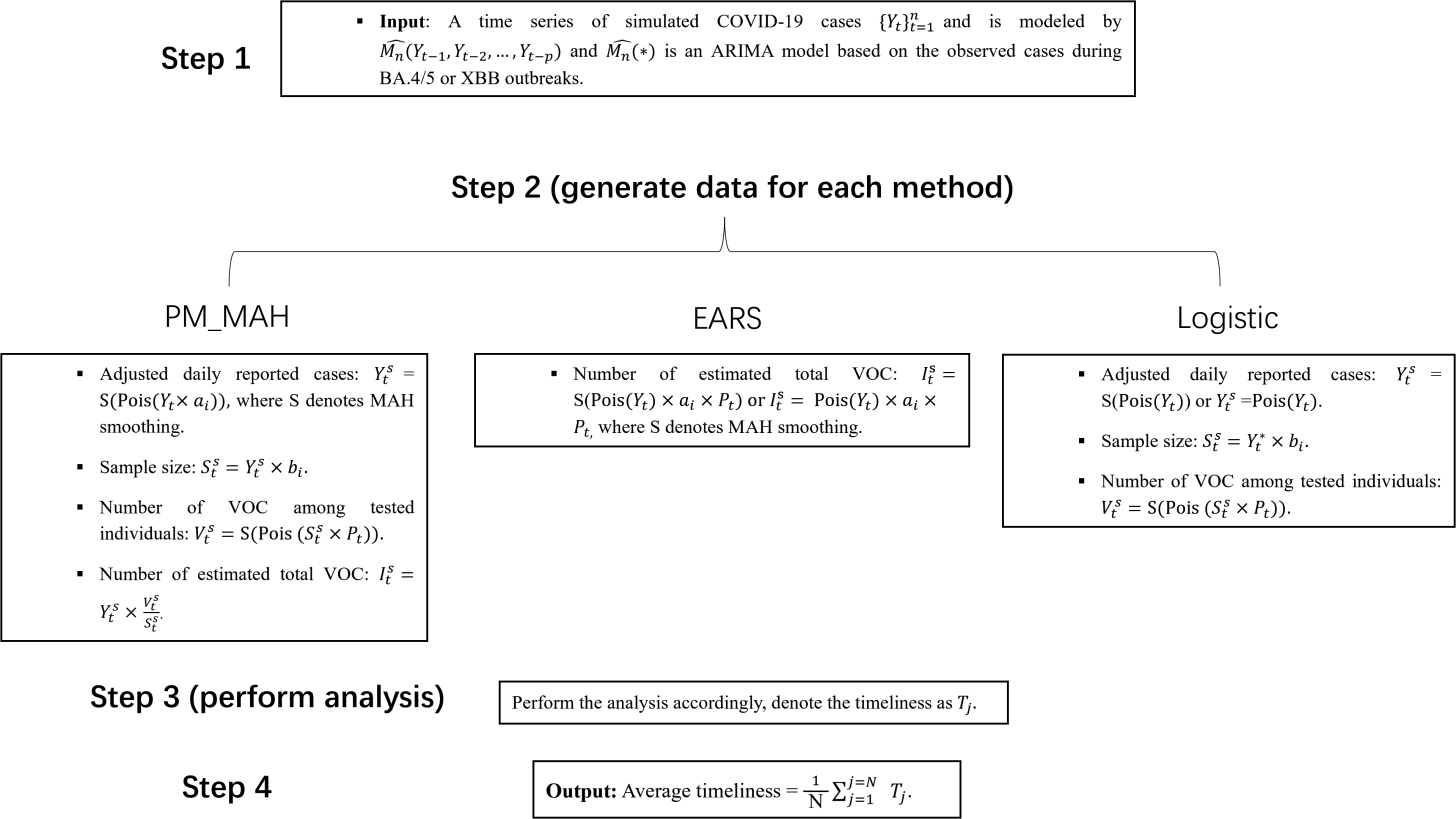
Steps of each method for simulation study 1.

Parameter *a* was varied to simulate different overall case volumes—representing scenarios from low-incidence to high-incidence surveillance periods—to examine how the magnitude of observed data influences model stability and detection performance. Similarly, parameter *b* was adjusted to evaluate how the proportion of tested individuals affects the sensitivity of each method under different levels of surveillance intensity. The above analysis allowed us to assess how these variations affected the performance of each method during the BA.4/5 and XBB variant waves. The steps for the simulation study, from iterations 1 to N, are outlined in Fig 2.

#### 2.3.2 Simulation analysis 2.

We carried out an additional simulation to evaluate the performance of the proposed outbreak detection methods (with MAH smoothing and no smoothing), all EARS methods, EpiEstim method and the logistic regression method. The comparison focused on their timeliness while ensuring that all methods operated at the same fpr. In the simulation, we use 3 models to compare the performance of various methods. Model 1 and 2 are based solely on observed total case counts (excluding the logistic regression method), while Model 3 is designed to assess performance in stratified analyses (including all methods). Please refer to the S1 Supplementary Materials for further details.

## 3 Results

### 3.1 Results for smoothing method

**Fig 3:**
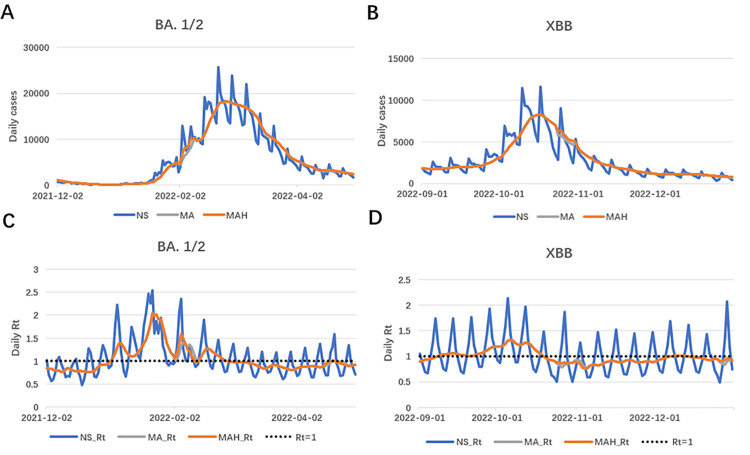
Panel A and B are adjusted cases for BA.1/2 and XBB outbreaks between different smoothing methods. Panel C and D are estimated Rt for BA.1/2 and XBB outbreaks between different smoothing methods.

As an illustration, Fig 3 compares various smoothing methods for the BA.1/2 and XBB waves. It is evident that the original data (NS) exhibited significant fluctuations due to the lack of adjustment of day-of-the-week and public holiday effects, with *R*_*t*_ oscillating around 1, making it difficult to detect outbreaks. In contrast, both the MA and MAH methods showed a smoother adjustment of cases, and *R*_*t*_ remained more stable, with the interference from calendar effects largely mitigated.

### 3.2 Comparison between outbreak detection methods for observed data

First, we selected the parameters for each method by controlling the fpr within a reasonable range. Considering that the serial interval for COVID-19 in Singapore varies between 2–4 days [30], we used constant generation times H of 2, 3, and 4 (as described in Section 2.2.2) and varied K from 1 to 4 (as detailed in Section 2.2.4) to evaluate the fpr of the proposed methods. The parameters for all methods were adjusted to ensure that the fpr remained at or below 0.01, 0.02, and 0.03.

**Fig 4:**
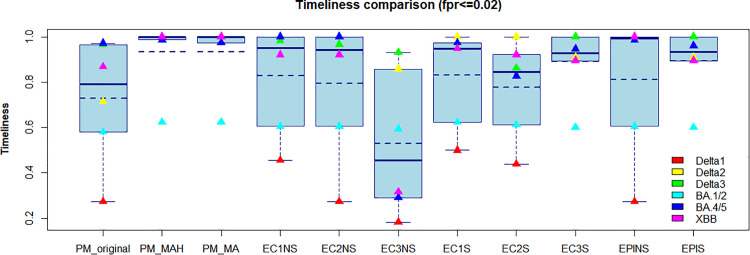
Comparing different methods in terms of timeliness, the EARS/EpiEstim methods that end with ’NS’ are based on the original data, while the EARS/EpiEstim methods that end with ’S’ are based on data smoothed using the MAH method. The solid navy-blue horizontal line represents the median timeliness for each method, whereas the dashed navy-blue line indicates the average timeliness.

Fig 4 illustrates the timeliness of various outbreak detection methods under fpr≤0.02. The solid navy-blue horizontal line indicates the median timeliness for each method, while the dashed line represents the average. Among the proposed methods (PM), PM_NS (K = 3) showed the poorest performance, as expected, due to the absence of smoothing on the input data. In contrast, PM_MA (K = 1) and PM_MAH (K = 1) yielded comparable results, with PM_MAH offering a slight edge. All EARS variants with the ‘NS’ suffix—based on unsmoothed data—performed worse than PM methods utilizing MA or MAH smoothing, particularly during the Delta 1 outbreak. During the BA.1/2 outbreak, however, all methods achieved similar timeliness scores. This likely reflects the relatively stable case counts between early December 2021 and mid-January 2022, a period of delayed case growth possibly influenced by Singapore’s phased reopening, including the introduction of vaccinated travel lanes, easing of event restrictions, and intensified contact tracing for BA.1 and BA.2 cases [31]. Consequently, all methods exhibited timeliness scores around 0.6 during this period. For more detailed results across different fpr thresholds, refer to the S1 Supplementary Materials, which shows consistent patterns. Notably, achieving fpr≤0.01 required substantially smaller *α* values for EARS C2 and C3, which severely compromised their performance. In contrast, PM_MAH, PM_MA, and EPIS continued to perform well under these stricter thresholds.

Although EARS methods do not rely on smoothing, we also applied them to MAH-smoothed data (suffix ‘S’). These smoothed variants exhibited moderate improvements in timeliness, and in several outbreaks achieved performance comparable to PM_MAH and PM_MA.

The EpiEstim method outperformed the EARS methods when applied to un-smoothed data but remained slightly less effective than both PM_MAH and PM_MA under fpr≤0.02 and fpr≤0.03. Although EpiEstim does not require smoothed input, we also assessed its performance using MAH-smoothed incidence. This smoothing notably improved timeliness during the Delta 1 outbreak but had limited effect on other outbreak waves.

Finally, the logistic regression method could not be directly evaluated in this comparison due to a key limitation: it requires differentiation between an emerging VOC and incumbent variants based on SGTF or WGS data. This requirement restricts its applicability to observed case counts alone.

### 3.3 Results for simulation analyses

#### 3.3.1 Results for simulation analysis 1.

**Fig 5:**
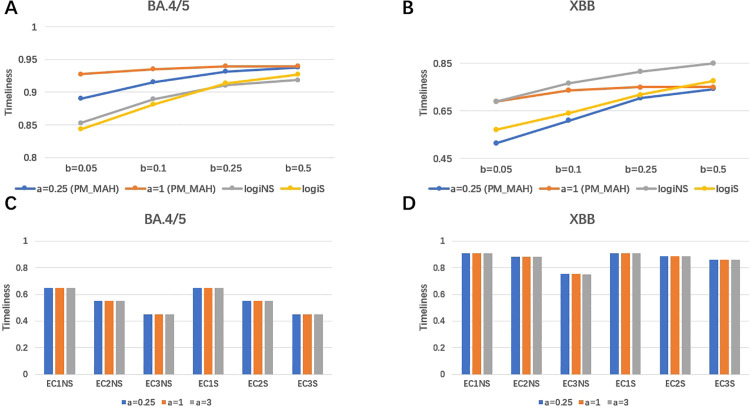
Panels A and B examine how the sample size of daily tested individuals (parameter b) and the total number of daily COVID-19 observations (parameter a) affect the performance of the proposed methods with MAH smoothing (PM_MAH), as well as the logistic regression method. In the logistic regression methods, those ending with ’NS’ are based on the original data, while those ending with ’S’ use data smoothed with MAH. Panels C and D evaluate the effect of total daily COVID-19 observations (parameter a) on the performance of the EARS methods. Similarly, EARS methods ending with ’NS’ use the original data, while those ending with ’S’ apply MAH smoothing. It should be noted that this analysis was not intended to compare timeliness across methods, but rather to evaluate how performance responds to changes in observation scale and sample size.

The simulation results for the BA.4/5 and XBB variants are presented separately in Fig 5, using the same parameter settings as in Fig 4 across all methods. This simulation analysis specifically evaluates the impact of sample size, expressed here as the daily fraction of individuals tested to determine the variant(parameter b, from 5% to 50%) and the total number of daily COVID-19 observations (parameter a, as a multiple of the observed number of cases, from 0.25 to 1.0). It is not intended to compare the timeliness of the methods directly, as they operate under different fpr. Consequently, direct comparisons of timeliness across methods are not appropriate.

In Fig 5 A and B, the findings indicate that increasing the fraction of daily tested individuals enhances timeliness for both the logistic regression method and the proposed method incorporating MAH smoothing. This result aligns with their theoretical properties, which will be discussed further in the Section 4. However, the timeliness of the proposed method plateaus beyond a certain threshold, as it can only generate signals starting from t=2H+K−1 or later.

Fig 5 C and D demonstrate the influence of the total number of daily COVID-19 observations on the performance of all EARS methods, which are based on the counts of all cases (i.e., without requiring testing to determine the variant). For these methods, timeliness remains largely unaffected as the total number of observed cases increases. This behavior is attributed to the structure of their test statistics and will be explained in more detail in the Section 4.

#### 3.3.2 Results for simulation analysis 2.

We began by adjusting the parameters for all methods across various fpr levels, except for the EARS C3 method with smoothing, as it could not achieve an fpr of 0.03 or lower. Once these adjustments were made, we evaluated the timeliness performance across all models.

**Fig 6:**
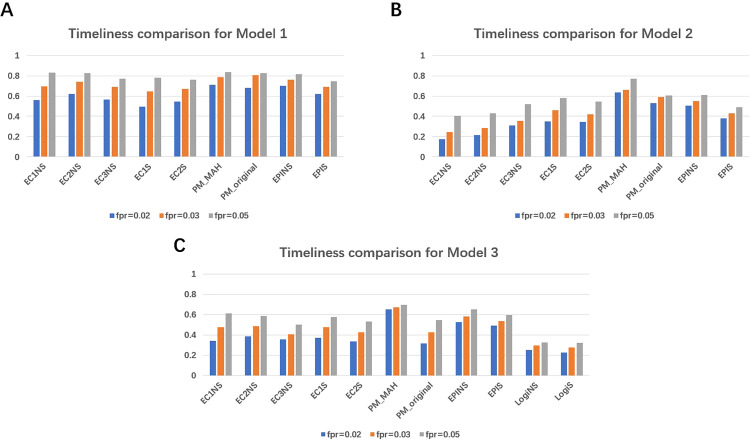
Each panel corresponds to a specific model and compares the timeliness of all methods while maintaining the false positive rate at the same level. For EARS methods, those ending with ’NS’ are based on the original data, while those ending with ’S’ use data smoothed with MAH. Model 1 and 2 rely solely on observed total cases (excluding the logistic regression model for comparison), with the difference being that the DGP in model 1 excluded while the DGP in model 2 included day-of-the-week and public holiday effects. Model 3 is designed to assess performance in stratified analyses (and hence include the logistic regression model), with day-of-the-week and public holiday effects are simulated as well. Further details are provided in the S1 Supplementary Materials.

As shown in Fig 6, for Model 1—where the DGP excluded day-of-the-week and public holiday effects—smoothing did not improve the timeliness of either the proposed methods or the EARS and EpiEstim approaches. This was expected, as the DGP lacked calendar-related anomalies. Nonetheless, the proposed method consistently outperforms all other methods by a small margin in terms of timeliness, regardless of whether smoothing is applied.

For Model 2, which included calendar effects and random noise, MAH smoothing moderately improved timeliness for both the proposed and EARS methods. In contrast, it had little impact on EpiEstim, likely because EpiEstim already applies temporal smoothing via its sliding time window. Overall, the proposed method with MAH smoothing consistently achieves the best timeliness, followed by EpiEstim without smoothing.

For Model 3—which introduced varying day-of-the-week and public holiday effects across different periods compared to Model 2—the results were mixed. While smoothing slightly improved the timeliness of EARS C1 at lower fpr levels, it had no effect on EARS C2, and the overall performance of the EARS methods remained limited under this more complex DGP. Similarly, the logistic regression method performed poorly, likely due to the modest growth advantage of the VOC over the non-VOC variant, which reduced its ability to detect differential growth. Smoothing also provided little benefit to this method, as it relies on relative growth rates that are minimally affected by calendar-driven fluctuations. EpiEstim’s performance remained largely unchanged with smoothing, consistent with findings in Model 2. In contrast, the proposed method consistently benefited from smoothing, with MAH substantially enhancing its timeliness. Notably, the MAH-smoothed version (PM_MAH) outperformed all other methods across all scenarios, followed closely by EpiEstim without smoothing (EPINS).

In conclusion, the simulation study highlights that MAH smoothing offers substantial benefits for both the proposed method and the EARS methods when calendar anomalies are present in the DGP, although the magnitude of these benefits varies across scenarios. Overall, the proposed method with MAH smoothing consistently emerged as the best performer, followed by the EpiEstim method—an expected outcome given the more sophisticated design of these two approaches.

## 4 Discussion

In this work, we confirmed the importance of smoothing methods [16] to adjust for the anomalies caused by day-of-the-week and public holiday effect in COVID-19 data reported in Singapore, spanning from 2021/04/01–2023/02/13. Employing these calendar-aware smoothing techniques go beyond improving visual representation of epidemic curves but also significantly stabilise effective reproduction number estimates. By integrating this adjustment directly into the outbreak detection framework, rather than treating it as a separate preprocessing step, we ensured that the estimation of *R*_*t*_ and subsequent inference were performed on calendar-stable inputs, reducing artefacts and improving interpretability.

While the effective reproduction number has long been a fundamental concept in explaining why epidemics occur, we showed that these stabilized estimates also allow it to be an effective indicator of when an outbreak is in progress. When used with smoothed case surveillance data, a proposed algorithm using the reproduction number outperformed several other statistical methods for flagging the start of COVID-19 epidemics in Singapore. Then to support more robust comparison under a variety of scenarios, we applied bootstrap methods [16–18] to draw statistical inference for effective reproduction number versus other methods, with and without smoothing. This provided a rigorous framework for assessing the timeliness and reliability of outbreak detection, and yielded insights on how smoothing of day-of-the-week and public holiday effects, as well as observed incidences and sample sizes affects the performance of different methods.

[8] highlighted the importance of addressing anomalies caused by day-of-the-week and public holidays to appropriately smooth healthcare data for infectious disease surveillance. We took their work one step further by showing how calendar-aware smoothing techniques significantly improves detection timeliness, both in established methods like EARS as well as our proposed method. Conceptually, calendar anomalies from the day-of-the-week are a source of “periodic noise” in surveillance data. Consequently, algorithms that neglect adjustment for these will require thresholds to manage false positive signals that also compromise timely outbreak detection. However, smoothing algorithms that account for such periodic effects by aggregating data over several days may also result in reduced timeliness. In this paper, we showed both in real data and a series of simulated scenarios, smoothing does improve the performance of most algorithms, as evidenced in the context of COVID-19 epidemics. Additional adjustment for public holidays slightly outperformed smoothing for day-of-the-week on its own. Public holidays are relatively rare, and the degree of improvement hence depends on when the holidays occur relative to critical time points in an epidemic. We would recommend to routinely adjust for this since our results show how the combined MAH algorithm best mitigates anomalies from calendar effects.

Having established the relevance of the smoothing algorithm, we tested the timeliness of our proposed method based on reproduction number as an early warning of impending COVID-19 epidemics. Results using both observed and simulated data for several established techniques [19,21,32] were also explored for comparison. Overall, the family of EARS methods exhibited varying performance across different scenarios. Within the EARS family, no single method consistently outperformed the others. In contrast, our proposed method (incorporating calendar-aware smoothing) demonstrated modest enhancements in timeliness compared to all EARS and EpiEstim methods. Taken together, integrating statistical and epidemiological components within a unified structure positions our framework as a practical extension of existing threshold- and regression-based detection systems. It achieves a balance between interpretability, sensitivity, and operational simplicity, making it broadly applicable to real-time surveillance settings.

Using simulation, for additional insights into their applicability to different scenarios, we also explored if the sample size of tested individuals and the incident number of observations choice affected the performance of the various methods. The EARS methods were minimally influenced by the number of incident cases in surveillance data, with the small fluctuations in timeliness for different case numbers in Fig 5 due to the resampling procedures rather than the number of simulated cases. A close examination of the EARS methods show that their test statistics utilize a standard normal distribution, and consequently their performance is minimally influenced by the number of incident cases in surveillance data.

To illustrate why this is so, let us set the baseline window size to *w* for EARS-C1. Assuming the case count is *Y*_*t*_, under the null hypothesis of “no outbreak”, the expected value of *Y*_*t*_ is approximated by the mean of the previous *w* observed case counts:


$${\bar{Y}}_{1}(t)=\frac{1}{w}\sum_{i=t-w}^{t-1}Y(i).$$

Additionally, the variance of *Y*_*t*_ is approximated by the sample variance of the previous *w* counts:


$$S_{1}^{2}(t)=\frac{1}{w-1}\sum_{i=t-w}^{t-1}[Y(i)-{\bar{Y}}_{1}(i){]}^{2},$$

which follows the distribution:


$$S_{1}(t)∼\sqrt{\frac{\sigma^{2}}{w-1}\chi_{w-1}^{2}},$$

where $\chi_{w}^{2}$ is a chi-squared random variable with *w* degrees of freedom.

Since the mean and variance are approximated by the previous *w* counts, under the null hypothesis of no outbreak, the following statistic was defined by EARS-C1:


$$C_{1}(t)=\frac{Y(t)-{\bar{Y}}_{1}(t)}{S_{1}(t)}∼N(0,1),$$

and an alarm is raised if $C_{1}(t)\geq Z_{1-\alpha }$, where $Z_{1-\alpha }$ is the critical value from the standard normal distribution. This statistic *C*_1_(*t*) is hence independent of the case count.

Our exposition of the statistics underlying the EARS method highlights a key issue: for the same relative change, outbreak signals based on data with larger case counts should generally be more reliable than those with smaller case counts. However, this difference in reliability is not reflected in the EARS methods. Although EARS allows for adjusting the fpr by modifying the *α* level, it fails to account for scenarios where there is insufficient training data to properly calibrate the fpr. These results are consistent with those shown in Fig 5, where the all EARS methods have the same timeliness results regardless of different total number of daily COVID-19 observations.

Compared to the EARS methods, EpiEstim offers several advantages for outbreak detection. As a model-based approach, EpiEstim estimates *R*_*t*_, capturing changes in transmission dynamics rather than relying solely on deviations from recent case counts. This allows for earlier detection of outbreaks, particularly when *R*_*t*_ exceeds 1, even before large increases in case numbers are observed. EpiEstim is also less sensitive to random fluctuations and day-of-week effects in the reported data, especially when combined with smoothing techniques (sliding window). Furthermore, it remains informative during low-incidence periods and provides uncertainty estimates through confidence intervals for *R*_*t*_, enhancing interpretability and supporting risk-based decision-making. In contrast, EARS relies on empirical thresholds and is more vulnerable to noise and delayed signals during the early stages of an outbreak.

A key limitation of the EARS method becomes apparent when applied to variant-specific data—a drawback also shared by EpiEstim. Both methods rely solely on observed case counts and do not account for the underlying sample size used to determine the variant. As a result, they generate identical signals regardless of whether the estimates are based on a sample of 50 or 500 individuals, potentially leading to misleading conclusions when sample sizes vary substantially. In contrast, the logistic regression method showed increasing timeliness with increasing sample size of tested individuals, which is also in-line with its theoretical property as the variance of the estimate—being the inverse of Fisher information—decreases as the sample size increases.

One obvious limitation of the proposed outbreak detection method is its inability to detect outbreaks right at the onset of epidemics, as it necessitates the exclusion of early observations when calculating *R*_*t*_. This limitation is shared by the EARS approach, which also requires some baseline data for effective detection. In contrast, the logistic regression method’s design allows it to function independently of prior data, enabling it to identify outbreaks at the onset of an epidemic when the new VOC has a strong growth rate relative to the incumbents. However, the method based on logistic regression requires that we have some form of laboratory testing to differentiate the new VOC from the incumbent variants against which it is being compared. In particular, if WGS is used to discriminate variants, this introduces an additional lag of several days between the point when samples are collected to when the data can be analysed.

It is also theoretically possible for the logistic regression method to result in a “false positive” of predicting an epidemic by a new VOC that is shrinking in incidence but is still growing relative to incumbent variants undergoing even more rapid decline. These complexities underscore the importance of selecting an appropriate method based on the specific epidemiological context, technical feasibility, and data constraints.

Finally, all methods will likely be susceptible to false positive signals when encountering exponential growth of imported cases of a variant to which the local population has herd immunity. This remains an untested scenario in the case of COVID-19 but could occur should a more effective vaccine be developed. Finally, the proposed method was validated here on case notification data. However, the method can potentially be applied to data from sentinel testing, which is available for COVID-19 in Singapore. This could be a focus for future extensions of this method, where comparisons would also need to be made against methods based on viral load indices from wastewater testing [33].

## 5 Conclusion

In this study, we addressed the significant impact of day-of-the-week and public holiday effects in syndromic data, highlighting the importance of these adjustments through analyses of COVID-19 data and comprehensive simulation studies. By integrating smoothing techniques with bootstrap resampling methods, we developed a quantitative framework for outbreak detection that performed consistently across both real and simulated outbreaks when compared with EARS and EpiEstim algorithms. It also has simplicity in terms of parameter choices, with the only key setting being the assumed serial interval, which can be informed by external literature. These are advantages which build confidence in its applicability to future events. In addition, given its theoretical foundation in the principle of the reproduction number, it may also work with other infections like influenza with similar transmission dynamics, and this should be an area for future work.

## Supporting information

S1 Text Additional analyses in this study. (PDF)
