# Supplementary material for: A smoothing and bootstrap-based framework for early outbreak detection
Source: PLoS One. 2026 Mar 23;21(3):e0345088. doi: 10.1371/journal.pone.0345088 (PMC13008254; doi:10.1371/journal.pone.0345088)
Supplement: S1 Text — (PDF) [file pone.0345088.s001.pdf]

S1 Supplementary materials for 'A smoothing and bootstrap-based framework for early outbreak detection'

Lengyang Wang[1], Yingcun Xia[2], Ee Hui Goh[1], and Mark Chen[3*]

[1] Advanced Methods and Analytics, Communicable Diseases Agency, Singapore

[2] Department of Statistics and Data Science, National University of Singapore, Singapore

[3] Department of Epidemiology and Preventive Medicine, Tan Tock Seng Hospital, Singapore

[*] Corresponding author(s). E-mail(s): mark_ic_chen@ttsh.com.sg

1 Timeliness results for Singapore COVID-19 data with different fpr levels

We also explored a regression-based approach to remove the day-of-the-week and public-holiday effects [1]. However, its performance was suboptimal, likely because the magnitude of each COVID-19 outbreak wave differed substantially, making it difficult for a simple regression model to consistently adjust for these calendar effects.

Let Y_t denote the daily number of COVID-19 cases. The model is expressed as

$$E(Y_t) = \beta_0 + \sum_{j\in\{\text{Tue},\dots,\text{Sun}\}} \beta_j^{(\text{dow})}\, I\{\text{DOW}_t = j\} + \beta_{\text{PH}}\, I\{\text{PH}_t = 1\} + \beta_{\text{FD}}\, I\{\text{FD}_t = 1\} + \sum_{k=1}^{5} f_k(Y_{t-k}),$$

where DOW is day of the week, PH is if the day is public holiday, FD is if the day is the first day after the public holiday and $f_k(\cdot)$ is a smooth function estimated from the data.

The estimated case counts after removing the day-of-the-week and public-holiday effects are obtained as

$$Y_t' = Y_t - \Big(\hat\beta_0 + \sum_{j\in\{\text{Tue},\dots,\text{Sun}\}} \hat\beta_j^{(\text{dow})}\, I\{\text{DOW}_t = j\} + \hat\beta_{\text{PH}}\, I\{\text{PH}_t = 1\} + \hat\beta_{\text{FD}}\, I\{\text{FD}_t = 1\}\Big).$$

We then performed the subsequent analysis using Y_t', referred to as the PM_REG method.

Table 1 summarizes the timeliness performance of each method across different false positive rate (fpr) thresholds. The model parameters were selected from the following candidate sets: $H \in \{2, 3, 4\}$ and $K \in \{1, 2, 3, 4, 5\}$. For each method, we report only the best-performing result across all combinations of H and K.

The start and peak dates of each outbreak are defined as follows:

- **Delta 1:** Start: 2021-05-04, Peak: 2021-05-15.[2]
- **Delta 2:** Start: 2021-07-12, Peak: 2021-07-19.[3]
- **Delta 3:** Start: 2021-08-29, Peak: 2021-10-26.[4]
- **BA.1/2:** Start: 2021-12-02, Peak: 2022-02-27. [5]
- **BA.4/5:** Start: 2022-05-04, Peak: 2022-07-12. [6]
- **XBB:** Start: 2022-09-09, Peak: 2022-10-17. [7]

Table 1: Comparison of methods under different fpr levels

Outbreak	PM_original	PM_REG	PM_MAH	PM_MA	EC1NS	EC2NS	EC3NS	EC1S	EC2S	EC3S	EPINS	EPIS
					**fpr $\leq$ 0.01**							
Delta1	0.182	0.000	0.438	0.438	0.455	0.273	0.091	0.500	0.438	0.313	0.273	1.000
Delta2	0.714	0.000	0.909	0.909	1.000	1.000	0.857	1.000	1.000	0.909	0.857	0.909
Delta3	0.948	0.839	0.983	0.983	0.983	0.948	0.931	0.172	0.138	0.121	1.000	1.000
BA.1/2	0.580	0.728	0.612	0.612	0.605	0.605	0.580	0.624	0.612	0.588	0.605	0.600
BA.4/5	0.971	0.913	0.973	0.973	1.000	0.812	0.174	0.827	0.813	0.800	0.986	0.960
XBB	0.868	0.816	0.974	0.974	0.921	0.553	0.289	0.921	0.921	0.868	0.921	0.895
**Mean**	**0.711**	**0.549**	**0.815**	**0.815**	**0.827**	**0.698**	**0.487**	**0.674**	**0.654**	**0.600**	**0.774**	**0.894**
					**fpr $\leq$ 0.02**							
Delta1	0.273	0.000	1.000	1.000	0.455	0.273	0.182	0.500	0.438	1.000	0.273	1.000
Delta2	0.714	0.000	1.000	1.000	1.000	1.000	0.857	1.000	1.000	0.909	1.000	0.909
Delta3	0.966	0.839	1.000	1.000	0.983	0.966	0.931	0.948	0.862	1.000	1.000	1.000
BA.1/2	0.580	0.728	0.624	0.624	0.605	0.605	0.593	0.624	0.612	0.600	0.605	0.600
BA.4/5	0.971	0.913	0.987	0.973	1.000	1.000	0.290	0.973	0.827	0.947	0.986	0.960
XBB	0.868	0.816	1.000	1.000	0.921	0.921	0.316	0.947	0.921	0.895	1.000	0.895
**Mean**	**0.729**	**0.549**	**0.935**	**0.933**	**0.827**	**0.794**	**0.528**	**0.832**	**0.777**	**0.892**	**0.811**	**0.894**
					**fpr $\leq$ 0.03**							
Delta1	0.273	0.000	1.000	1.000	0.455	0.273	0.182	0.500	1.000	1.000	0.273	1.000
Delta2	0.714	0.125	1.000	1.000	1.000	1.000	0.857	1.000	1.000	0.909	1.000	1.000
Delta3	0.966	0.910	1.000	1.000	0.983	0.966	0.948	0.966	1.000	1.000	1.000	1.000
BA.1/2	0.580	0.777	0.624	0.624	0.605	0.605	0.593	0.624	0.624	0.600	0.605	0.600
BA.4/5	0.971	0.956	0.987	0.973	1.000	1.000	0.986	0.987	0.973	0.947	0.986	0.973
XBB	0.868	0.868	1.000	1.000	0.921	0.921	0.526	1.000	0.947	0.895	1.000	0.921
**Mean**	**0.729**	**0.606**	**0.935**	**0.933**	**0.827**	**0.794**	**0.682**	**0.846**	**0.924**	**0.892**	**0.811**	**0.916**

2 More details for simulation study 2

For Model 1, the observed total cases $\{X_t\}_{t=1}^{260}$ are simulated as:

$$
\begin{aligned}
Y_t &= \begin{cases} 0.55 \times Y_{t-1} + 0.3 \times Y_{t-2} + \varepsilon_t + 2, & \text{if } t \leq 210, \\ 0.55 \times Y_{t-1} + 0.3 \times Y_{t-2} + \varepsilon_t + 2.5, & \text{if } t > 210, \end{cases} \\
X_t &= Y_t \times 40.
\end{aligned}
$$

For Model 2, the observed total cases $\{X_t\}_{t=1}^{260}$ are simulated as:

$$
\begin{aligned}
Y_t &= \begin{cases} 0.55 \times Y_{t-1} + 0.3 \times Y_{t-2} + \varepsilon_t + 2, & \text{if } t \leq 130, \\ 0.55 \times Y_{t-1} + 0.3 \times Y_{t-2} + \varepsilon_t + 2 \times 0.96^{(t-130)/3}, & \text{if } 130 < t \leq 210, \\ 0.55 \times Y_{t-1} + 0.3 \times Y_{t-2} + \varepsilon_t + 0.67 \times 1.05^{(t-210)/3}, & \text{if } t > 210, \end{cases} \\
Y_t &= Y_t + 3, \quad \text{if (t mod 10 = 0) and } 80 \leq t \leq 210, \\
Y_t &= Y_t + \frac{1}{Z_t}, \\
X_t &= Y_t \times 5.
\end{aligned}
$$

Here, $\varepsilon_t \sim N(0, 0.25)$ and $Z_t \in \{1, 2, 3, 4, 5, 6, 7\}$ indicates the day of the week. To model public holiday effects, we also set $Z_t = 7$ if $t \mod 30 = 0$. We add some random noises when $80 \leq t \leq 210$, as the effects of day-of-the-week and public holidays can vary, and there may be under-reporting issues on specific dates. When $t \leq 210$:

- In Model 1, which represents a stationary time series, no outbreak signals should be detected; any signals identified during this period would be considered false positives.

- In Model 2, the time series is non-stationary as a result of calendar anomalies and random noise. Despite this, we maintain the assumption that no outbreak signals should be detected since there is no increasing trend of the cases.

When $t > 210$, the presence of a genuine increase in the mean indicates that any detected outbreak signals are valid for both models.

For Model 3, the observed total cases $\{X_t\}_{t=1}^{260}$, number of people taking tests

$\{S_t\}_{t=1}^{260}$, and number of VOC among tested people $\{V_t\}_{t=1}^{260}$ are simulated as:

$$Y_t = \begin{cases} 0.55 \times Y_{t-1} + 0.3 \times Y_{t-2} + \varepsilon_t + 2, & \text{if } t \leq 130, \\ 0.55 \times Y_{t-1} + 0.3 \times Y_{t-2} + \varepsilon_t + 2 \times 0.96^{(t-130)/3}, & \text{if } 130 < t \leq 210, \\ 0.55 \times Y_{t-1} + 0.3 \times Y_{t-2} + \varepsilon_t + 0.67 \times 1.05^{(t-210)/3}, & \text{if } t > 210, \end{cases}$$

$$B_t = \begin{cases} 0.55 \times B_{t-1} + 0.3 \times B_{t-2} + \varepsilon_t + 2, & \text{if } t \leq 130, \\ 0.55 \times B_{t-1} + 0.3 \times B_{t-2} + \varepsilon_t + 2 \times 0.96^{(t-130)/3}, & \text{if } 130 < t \leq 210, \\ 0.55 \times B_{t-1} + 0.3 \times B_{t-2} + \varepsilon_t + 1 \times 1.05^{(t-210)/3}, & \text{if } t > 210, \end{cases}$$

$$S_t = Y_t \times 3,$$

$$Y_t = Y_t + \frac{2}{Z_t}, \quad B_t = B_t + \frac{2}{Z_t}, \quad \text{if } t \leq 130,$$

$$Y_t = Y_t + \frac{4}{Z_t}, \quad B_t = B_t + \frac{4}{Z_t}, \quad \text{if } 130 < t \leq 210,$$

$$Y_t = Y_t + \frac{1}{Z_t}, \quad B_t = B_t + \frac{1}{Z_t}, \quad \text{if } t > 210,$$

$$X_t = Y_t \times 10,$$

$$V_t = B_t \times 0.6.$$

Parameters ε_t have the same distributions as those in Model 1 and 2. Model 3 removes the random noises compared to Model 2 but lets day-of-the-week and public holiday effects change in different periods. When $t > 210$, the growth rate of the VOC surpasses that of the non-VOC. In this period, the presence of a genuine increase in the mean indicates that any detected outbreak signals are valid.

3 More discussions about the proposed method

Without loss of generality, we set $H = 1$. As previously mentioned, we assume the cases follow Poisson distributions:

$$Y_t \sim \text{Poisson}(\lambda_t), \quad t \in \{1, 2, \dots\}.$$

At time t, consider the following hypothesis testing problem:

$$H_0 : R_t \leq 1 \quad \text{vs.} \quad H_1 : R_t > 1.$$

If the observed value for Y_{t-1} is $\widetilde{\lambda}_{t-1}$, then under H_0 and with a normal approximation, the distribution of R_t at time t is:

$$R_t = \frac{Y_t}{\widetilde{\lambda}_{t-1}} \sim N\left(\frac{\lambda_t}{\widetilde{\lambda}_{t-1}}, \frac{\lambda_t}{\widetilde{\lambda}_{t-1}^2}\right) = N\left(\frac{\lambda_{t-1}}{\widetilde{\lambda}_{t-1}}, \frac{\lambda_{t-1}}{\widetilde{\lambda}_{t-1}^2}\right). \tag{1}$$

We reject H_0 if estimated R_t based on observed value is larger than cv_α, where $cv_\alpha > 0$ is the critical value determined by:

$$\text{pr}(R_t > cv_\alpha) = \alpha,$$

under H_0, with $\alpha \in (0, 1)$ being the significance level of the test. To determine cv_α, we need to derive the distribution of R_t under H_0, which requires the distribution of Y_{t-1}. As mentioned in the main paper, the conditional expectation

$$E(Y_{t-1} \mid Y_{t-2}, \ldots, Y_{t-p-1}),$$

can be modeled using a parametric estimator (e.g., ARIMA):

$$\mathcal{M}_n(Y_{t-2}, \ldots, Y_{t-p-1}).$$

Thus, based on observed data $\{Y_t\}_{t=1}^n$, we estimate λ_{t-1} as:

$$\hat{Y}_{t-1} = \hat{\lambda}_{t-1} = \widehat{\mathcal{M}}_n(Y_{t-2}, \ldots, Y_{t-p-1}). \tag{2}$$

This allows us to evaluate cv_α by drawing bootstrap samples R_t^*, where:

$$R_t^* = \frac{\text{Poisson}(\hat{\lambda}_{t-1})}{\widetilde{\lambda}_{t-1}},$$

with $\hat{\lambda}_{t-1}$ being an appropriate estimator for λ_{t-1} derived from an ARIMA model for $\mathcal{M}_n(*)$. Suppose $c\hat{v}_\alpha$ is the critical value obtained using the bootstrap method under H_0. Then, under H_0, we have:

$$\text{pr}(R_t > c\hat{v}_\alpha) = \alpha.$$

From (1), $\text{pr}(R_t > c\hat{v}_\alpha)$ can be expressed as:

$$\text{pr}\left(R_t > \frac{\lambda_{t-1}}{\widetilde{\lambda}_{t-1}} + Z_{1-\alpha}\sqrt{\frac{\lambda_{t-1}}{\widetilde{\lambda}_{t-1}^2}}\right) = \text{pr}\left(\frac{R_t - \frac{\lambda_{t-1}}{\widetilde{\lambda}_{t-1}}}{\sqrt{\frac{\lambda_{t-1}}{\widetilde{\lambda}_{t-1}^2}}} > \frac{\left(\frac{\lambda_{t-1}}{\widetilde{\lambda}_{t-1}} + Z_{1-\alpha}\sqrt{\frac{\lambda_{t-1}}{\widetilde{\lambda}_{t-1}^2}}\right) - \frac{\lambda_{t-1}}{\widetilde{\lambda}_{t-1}}}{\sqrt{\frac{\lambda_{t-1}}{\widetilde{\lambda}_{t-1}^2}}}\right),$$
$$= \text{pr}(Z > Z_{1-\alpha}) = \alpha,$$

where Z is a standard normal variable.

Now suppose there is a mean increase at time t, such that $\mu(Y_t) = \lambda_t > \mu(Y_{t-1}) = \lambda_{t-1}$. If the observed value of $Y_{t-1} = \widetilde{\lambda}_{t-1}$, the distribution of R_t under H_1 is:

$$R_t = \frac{Y_t}{\widetilde{\lambda}_{t-1}} \sim N\left(\frac{\lambda_t}{\widetilde{\lambda}_{t-1}}, \frac{\lambda_t}{\widetilde{\lambda}^2_{t-1}}\right). \tag{3}$$

Similarly, under H_1, we have:

$$\mathrm{pr}(R_t > c\hat{v}_\alpha) = 1, \quad \lambda_t \to \infty.$$

From (1) and (3), $\mathrm{pr}(R_t > c\hat{v}_\alpha)$ can be rewritten as:

$$\begin{aligned}
\mathrm{pr}\left(R_t > \frac{\lambda_{t-1}}{\widetilde{\lambda}_{t-1}} + Z_{1-\alpha}\sqrt{\frac{\lambda_{t-1}}{\widetilde{\lambda}^2_{t-1}}}\right) &= \mathrm{pr}\left(\frac{R_t - \frac{\lambda_t}{\widetilde{\lambda}_{t-1}}}{\sqrt{\frac{\lambda_t}{\widetilde{\lambda}^2_{t-1}}}} > \frac{\left(\frac{\lambda_{t-1}}{\widetilde{\lambda}_{t-1}} + Z_{1-\alpha}\sqrt{\frac{\lambda_{t-1}}{\widetilde{\lambda}^2_{t-1}}}\right) - \frac{\lambda_t}{\widetilde{\lambda}_{t-1}}}{\sqrt{\frac{\lambda_t}{\widetilde{\lambda}^2_{t-1}}}}\right), \\
&= \mathrm{pr}\left(Z > \frac{\left(\lambda_{t-1} + Z_{1-\alpha}\sqrt{\lambda_{t-1}}\right) - \lambda_t}{\sqrt{\lambda_t}}\right).
\end{aligned} \tag{4}$$

As seen from the RHS of (4), when $\lambda_t \to \infty$, the RHS of (4) approaches $-\infty$. Thus, it is evident that:

$$\mathrm{pr}(R_t > c\hat{v}_\alpha) = 1, \quad \lambda_t \to \infty.$$

References

[1] Xia, Y. and Tong, H., "Cumulative effects of air pollution on public health", *Statistics in Medicine*, 25 (2006), 3548–3559.

[2] Lai, L., "Singapore's largest active Covid-19 cluster: What went wrong at Changi Airport?", *The Straits Times* (2021).

[3] Zhang, L. M., "KTV and Jurong Fishery Port Covid-19 clusters linked: Ong Ye Kung", *The Straits Times* (2021).

[4] Lee, Y. N., "Singapore to start relaxing Covid restrictions Aug. 10 as vaccination rate rises", *CNBC* (2021).

[5] Chua, F. J. D. et al., "Co-incidence of BA.1 and BA.2 at the start of Singapore's Omicron wave revealed by Community and University Campus wastewater surveillance", *Science of The Total Environment*, 875 (2023), 162611.

[6] Leow, A., "A timeline of the Tan Tock Seng Hospital COVID-19 cluster", *Business Times* (2022).

[7] Pung, R. et al., "Severity of SARS-CoV-2 Omicron XBB subvariants in Singapore", *The Lancet Regional Health – Western Pacific*, 37 (2023).
